# Epidemiology of Multiple Congenital Anomalies Before and After Implementation of a Nationwide Prenatal Screening Program in Denmark

**DOI:** 10.3389/fped.2021.614864

**Published:** 2021-02-05

**Authors:** Marlene E. Toxværd, Ester Garne

**Affiliations:** Paediatric Department, Hospital Lillebaelt, Kolding, Denmark

**Keywords:** multiple congenital anomalies, EUROCAT, prenatal screening, prevalence, survival

## Abstract

Surveillance of congenital anomalies is important in order to detect negative influences from environment, medication, or lifestyle as early as possible. Since most teratogens are associated with a spectrum of birth defects rather than a single defect, analysis of the epidemiology of multiple congenital anomalies is important to detect an increase due to environmental or medicine exposure. The aim of the study was to describe changes in prevalence, types of anomalies, and outcome of pregnancies for fetuses and infants with multiple congenital anomalies before and after introduction of the new screening program in the County of Funen, Denmark. The study was based on data from the EUROCAT registry of the County of Funen for the period 1990 to 2014 covering 135,057 births. The registry includes information about livebirths, fetal deaths after 20 weeks of gestation and terminations of pregnancy after prenatal diagnosis of fetal anomalies. All cases with two or more major congenital anomalies in different organ systems, where the pattern of anomalies were not recognized as part of a chromosomal or genetic syndrome or a sequence were included in the study. Overall prevalence of multiple congenital anomalies was 19.7 per 10,000 pregnancies. There was no significant change in prevalence over time. The prenatal detection rate increased from 26 to 57% after introduction of the screening program (*p* < 0.001). Proportion of terminations of pregnancy increased from 11 to 30% of all cases and 1-week survival for livebirths increased from 64 to 94%. There was no change in combinations of involved organ systems. The implementation of the new screening program in 2004 has led to an increased prenatal detection rate of multiple congenital anomalies followed by an increased rate of termination of pregnancy for the most severe cases and an increased 1-week survival for liveborn infants with multiple congenital anomalies.

## Introduction

Surveillance of congenital anomalies has become an important public health activity since the thalidomide disaster, aiming to prevent a similar or smaller incidents (1). Surveillance of congenital anomalies is also of significant importance in order to detect negative influences from environment, medication, or lifestyle as early as possible and in this way prevent congenital anomaly clusters due to new teratogenic exposures (1). Since some teratogens are associated with a spectrum of birth defects rather than a single defect, analysis of the epidemiology of multiple congenital anomalies is important to detect an increase of those due to environmental or medicine exposure (2). Due to the rarity of multiple congenital anomalies, dataset over many years is necessary. Therefore, only very few studies have been dealing with surveillance of congenital anomalies leading to an unknown magnitude of the problem.

In 2004 Denmark replaced its earlier prenatal screening program, which included offering an invasive test to pregnant women over 34 years, with a screening program offered to all pregnant women. The new national screening program includes a nuchal translucency scan and a blood sample at a gestational age between the 11th and 13th week and an anomaly scan between the 18th and 20th week (3). The change was based on a recommendation from a working group set up by the Danish Ministry of Health. The recommendation suggested that by screening all pregnant women with a blood test and an ultrasound, the number of amniocentesis would decrease and simultaneously the prenatal detection rate of congenital anomalies would increase. At that time amniocentesis leads to a miscarriage in about 1 of 100 pregnancies, reducing the numbers was desirable and non-invasive methods should be preferred (3).

Detecting congenital anomalies during pregnancy has many advantages, such as the possibility of referring the mother before birth to a tertiary hospital with the required expertise for delivery, the possibility of giving the parents relevant information and preparation for the life with their infant and in severe cases providing an opportunity to offer termination of the pregnancy.

The aim of this study is to describe the influence of the screening program implemented in Denmark during 2004 on prenatal detection rates, rates of terminations of pregnancy and the impact on survival of liveborn infants with multiple congenital anomalies.

## Materials and Methods

EUROCAT is a European network of population-based registries for the epidemiological surveillance of congenital anomalies with more than 1.7 million births surveyed per year in Europe since 1980 (4). This study was based on data from the EUROCAT registry of Funen County, Denmark. The registry includes livebirths, fetal deaths from GA 20 weeks and terminations of pregnancy after prenatal diagnosis of congenital anomalies. Data from 1990 to 2014 was included, covering a population of 135,057 births (live and stillbirths) in the registry area.

Multiple congenital anomaly cases were defined as two or more major structural congenital anomalies in different organ systems. The cases were classified for this study using the EUROCAT Multiple Congenital Anomaly Algorithm (5). Cases with skeletal dysplasias and teratogenic, chromosomal, and genetic syndromes were excluded as well as all cases with anomalies in one organ system only (1). The remaining potential multiple congenital anomaly cases (*n* = 331) were manually evaluated by the authors, excluding cases with anomalies in one organ system only, as well as cases with genetic or chromosomal syndromes which were not picked up by the algorithm.

For the statistical evaluation MATLAB's Statistics and Machine Learning Toolbox was used. Descriptive data is presented in percentage. Trends over time were calculated using logistic regression in single years and presented in five periods (1990–1994, 1995–1999, 2000–2004, 2005–2009, 2010–2014). Logistic regression was also used for comparison of groups before and after introduction of general screening. For the evaluation of the effect of the new screening program, data from before implementation (1990–2004) was compared to data after implementation of the new screening (2005–2014). For all statistical test a significance level of 5% was used.

## Results

During the years 1990–2014 there were 265 fetuses and infants with multiple congenital anomalies, giving an overall prevalence of 19.7 per 10,000 births.

**Figure 1:**
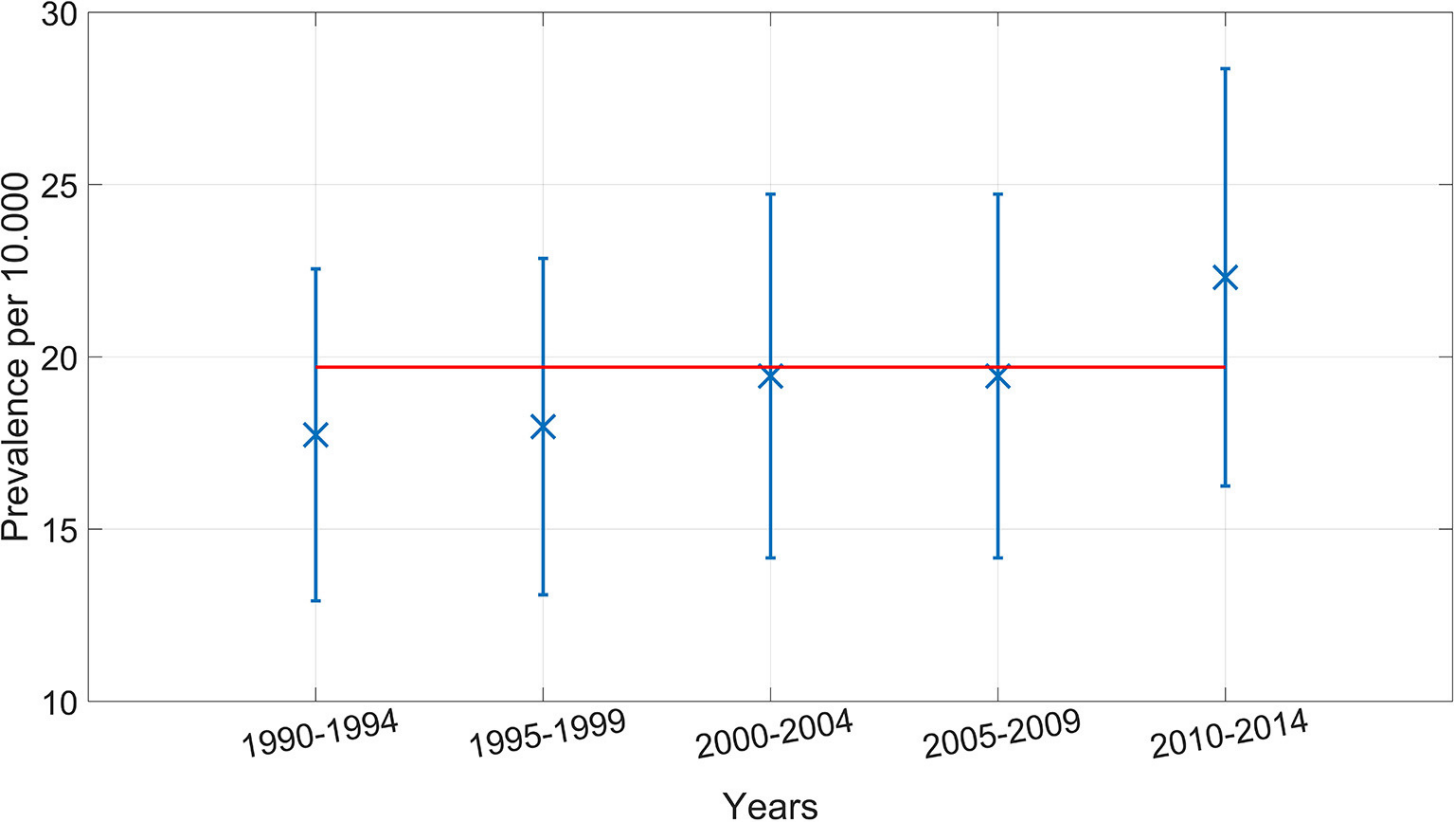
Prevalence of fetuses and infants with multiple anomalies per 5 year periods, Funen Denmark 1990–2014.

Prevalence of multiple congenital anomalies in the 15 years before implementation of the screening program was 18.5 per 10,000 births and increased to 21.6 per 10,000 births in the 10 years after the implementation (*p* = 0.21; Table 1). The prevalence in 5-year periods from 1990 to 2014 is presented in Figure 1. The observed increase in prevalence during these 25 years was not statistically significant.

**Table 1:** Prevalence of multiple congenital anomalies, prenatal detection rates, and outcomes of pregnancy.

	**1990–2004**	**2005–2014**	
Total births	84,998	50,059	
Cases with MCA	157	108	
Prevalence of MCA per 10,000 births	18.5	21.6	*p* = 0.21
Prenatal detection rate (N)	26% (41)	57% (62)	*p* < 0.001
Median GA at discovery, weeks	21	20	*p* < 0.05
Number (%) liveborn infants	127 (81%)	71 (66%)	*p* < 0.01
Number (%) of liveborn infants alive at 1 week	81 (64%)	67 (94%)	*p* < 0.001
Number (%) termination of pregnancy	17 (11%)	32 (30%)	*p* < 0.001
Number (%) fetal deaths from GA 20 weeks	13 (8 %) (13)	5 (4%)	*p* = 0.25

*MCA, multiple congenital anomaly*.

The prenatal detection rate increased from 26% before to 57% after implementation of the screening program (*p* < 0.001). Median GA at birth for liveborn infants with multiple congenital anomalies was 38 weeks in 1990–2004 and 39 weeks in 2005–14 (*p* = 0.43). In 1990–2004 infants with a prenatal diagnosis were born almost 2 weeks earlier than infants with a postnatal diagnosis (*p* = 0.05). This difference could not be observed in 2005–14. The introduction of nationwide prenatal screening lead to a mean GA at discovery of multiple anomalies at week 20, which was 3 weeks earlier than it used to be (*p* < 0.01), but the mean gestational age for termination did not change significantly, being 18.9 gestational weeks in the first period and 18.5 in the second period.

**Figure 2:**
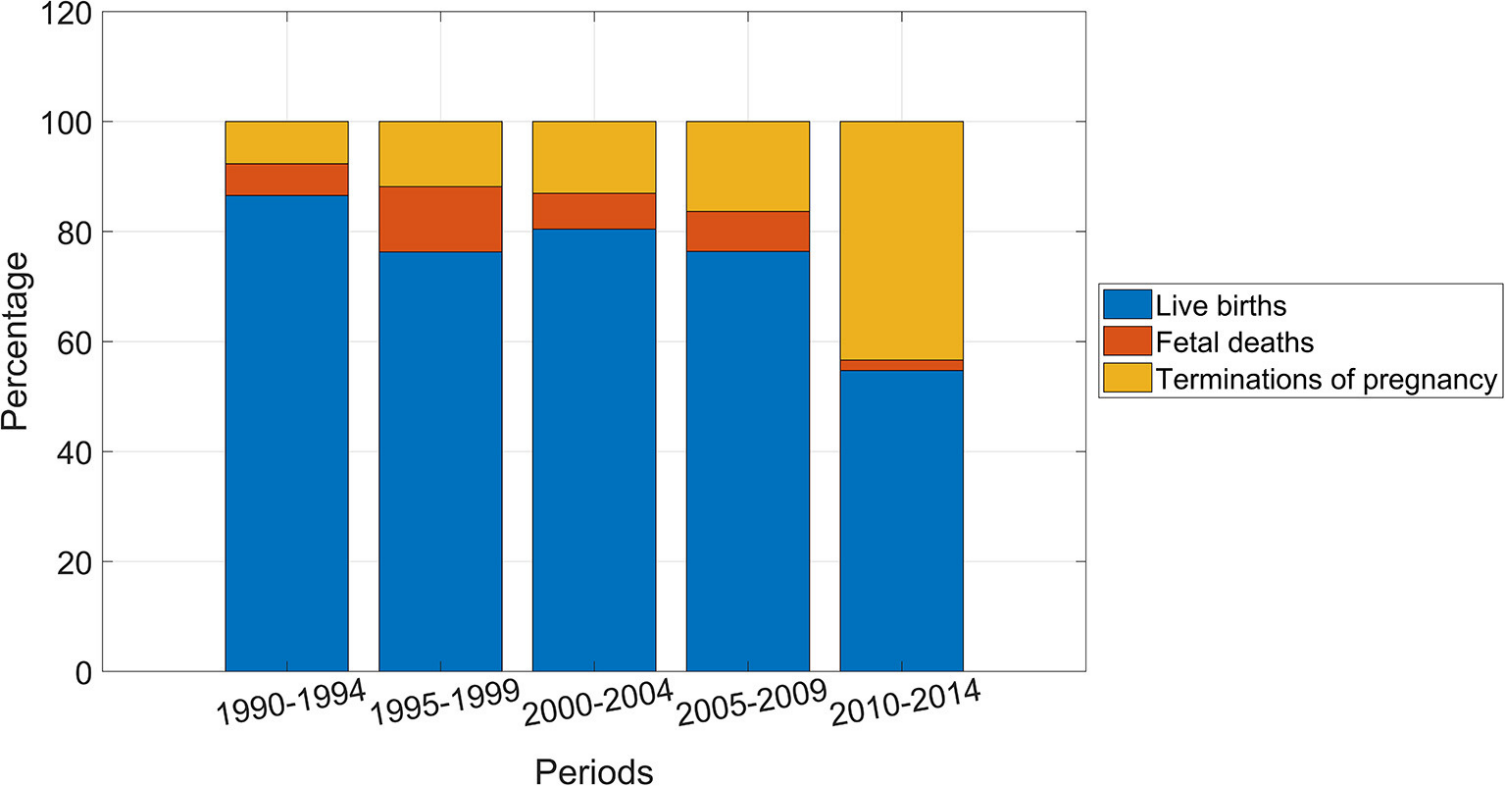
Distribution of pregnancy outcome for fetuses and infants with multiple congenital anomalies, Funen, Denmark, 5 year periods 1990–2014.

The distribution of the pregnancy outcomes in 5 year periods is shown in Figure 2. Proportion of terminations of pregnancy increased over time and proportion of livebirths decreased (Table 1).

First week survival of livebirths was 64% in the 15 years before implementation of the screening program and increased to 94% in the in the 10 years after the implementation (*p* < 0.01).

**Figure 3:**
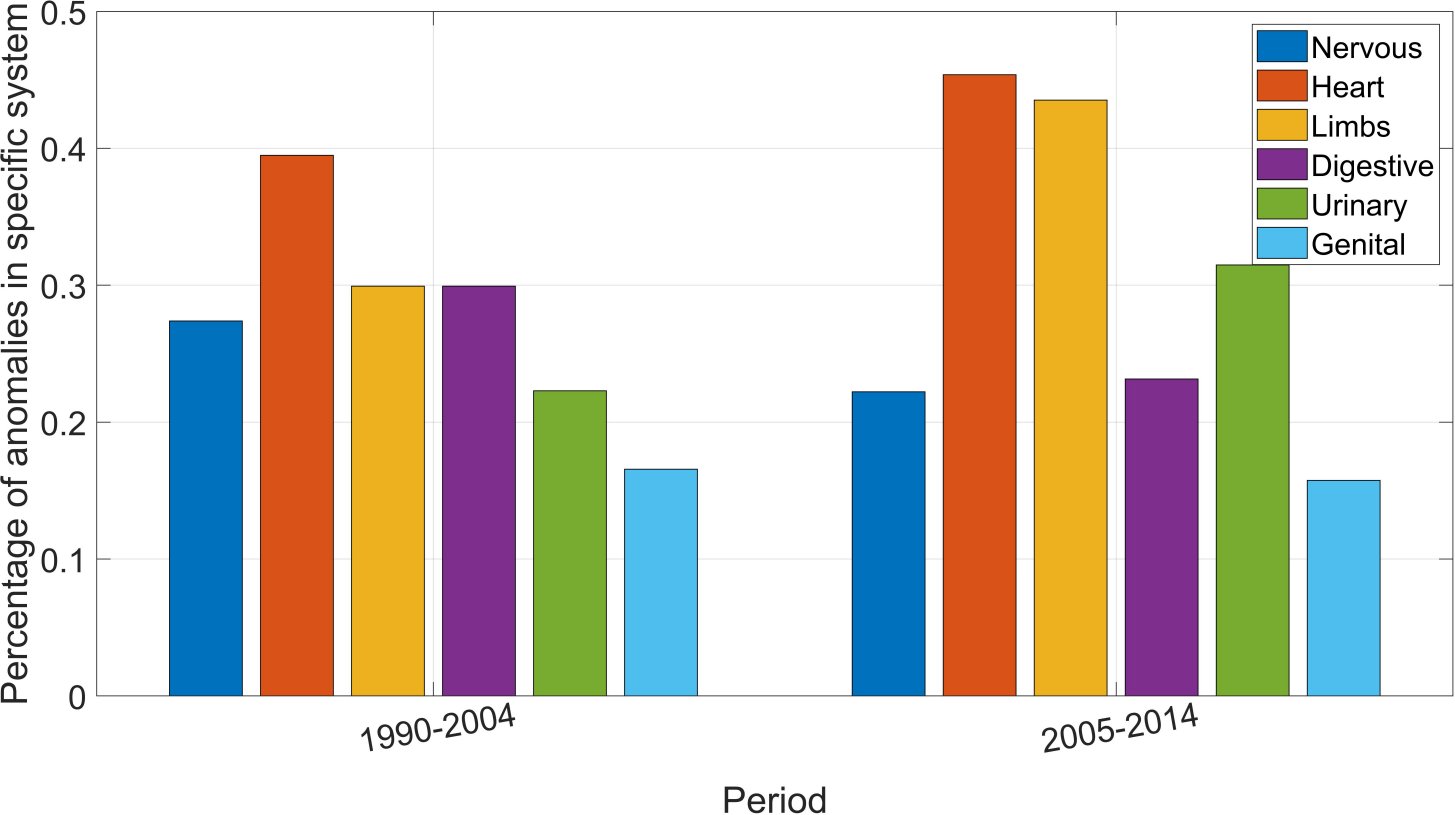
Percentage of congenital anomalies in respective organ system.

As shown in Figure 3 anomalies of the heart were the most common in both time periods (39% of all anomalies before 2005 and 46% in the later years), followed by limb defects. There has been an increase of anomalies of the urinary tract and simultaneous a reduction of anomalies of the digestive system, which is not statistically significant (*p* = 0.1). Anomalies of the eye, the face, the neck, and the abdominal wall were very rare in both periods with less than two cases in each period (not shown in Figure 3). The most common combination of anomalies were defects in the digestive system and the heart in both periods followed by defects of the heart and the urinary system with no significant change over time.

## Discussion

Surveillance of multiple congenital anomalies is important in order to detect any increase or clustering, considering that most teratogens are associated with a spectrum of anomalies rather than one specific anomaly.

Our study showed that the prevalence of fetuses and infants with multiple anomalies during the 25 years was 19.7 per 10,000 births. The prevalence published in the literature ranges from 15.1 per 10,000 births (6) to 16.3 per 10,000 births (7) and to 26.3 per 10,000 births (8). The given prevalence for Funen is comparable to these other studies. However, there may be different registration of cases between populations studied and differences in lifestyle/exposure patterns (1).

The study confirmed that the introduction of the nationwide screening program led to an increasing prenatal detection rate of multiple anomalies at an earlier gestational age. It furthermore showed that the prenatal detection has led to an increase in the termination rate, as nearly 30% of fetuses with multiple anomalies diagnosed prenatally were terminated in the recent time period. In the literature a prenatal detection rate between 51 and 58% has been found (6), which is very similar to the detection rate in this study after introduction of the nationwide screening program.

The study showed an increase in the 1-week survival rate, which indicate that the increase in the termination rate was mainly for fetuses with severe anomalies that would not have survived the 1st week. There was an increase of both survival rate and termination of pregnancy rate through all the years, but with an additional upturn seen after 2004. The increase of survival in the first period might be explained by improvements in diagnostic and treatment options in neonatology and intensive care in the early 90's and has also been described worldwide for overall neonatal survival (9). In Western Europe the neonatal mortality rate decreased by 58% from 1990 to 2017. Other published data showed an increase in overall neonatal survival from 96.7% in 1990 to 98% in 2013 (10). The increase in the termination rate might be explained by a more liberal attitude toward termination of pregnancy (11) and improved prenatal detection rates. Interestingly, in our study the gestational age at termination of pregnancy did not decrease significantly, although the gestational age at prenatal detection decreased. It might be due to small numbers in our study with only 17 terminations of pregnancies in the first 15 years of the study. The congenital anomalies detected before the nationwide screening program was introduced, were mainly severe anomalies that were easily detectable, such as anencephaly. There was a very small number of fetal deaths with multiple congenital anomalies in both periods with <2 fetal deaths per year.

Prenatal detection of major congenital anomalies requiring surgery in the neonatal period may lead to induced birth at an earlier GA than if spontaneous labor was awaited. In this study we found no significant change in gestational age of live born infants with multiple anomalies before and after introduction of the screening.

The distribution of the organ systems in the fetuses and infants with multiple anomalies was similar in both periods, except for an increase of defects of the urinary tract, and a decrease of defects of the digestive system. The prenatal screening program seems to have diagnosed some urinary anomalies that may have been undiagnosed without the screening program (congenital hydronephrosis and multicystic renal dysplasia). The distribution of organ systems involved is comparable to a study including 19 European countries (6).

### Strengths and Limitations

A major strength of the study is the high rate of participation of the screening program, in 2014 about 95% of all pregnant women participated (12).

Furthermore, the congenital anomalies data has been collected routinely over 25 years from the same data sources which makes the data comparable over time. The data collection has followed a strict algorithm in all years, enhancing comparability and data quality.

A limitation of the study is the rareness of the diseases, which makes it difficult to give a full comprehension of the influence of the screening procedure and a less accurate statistical analysis. Further, the diagnosis of additional congenital anomalies after the first diagnosis may differ for terminations of pregnancy compared to liveborn infants. Postmortem examinations was not performed for all terminations in the population.

## Conclusion

Implementation of a nationwide screening program in Denmark has led to an increasing prenatal detection rate of multiple congenital anomalies and to an increase in the rate of terminations of pregnancy for fetuses with multiple anomalies. Consequently, the 1-week survival rate for liveborn infants with multiple congenital anomalies has increased and most parents were prepared for having a baby with congenital anomalies and prepared for the necessary treatment. There was no significant change in the organ systems involved in the multiple congenital anomalies.

## Data Availability Statement

The datasets presented in this article are not readily available because individual data cannot be shared. Requests to access the datasets should be directed to ester.garne@rsyd.dk.

## Author Contributions

All authors listed have made a substantial, direct and intellectual contribution to the work, and approved it for publication.

## Conflict of Interest

The authors declare that the research was conducted in the absence of any commercial or financial relationships that could be construed as a potential conflict of interest.
